# Stabilization of the c-Myc Protein via the Modulation of Threonine 58 and Serine 62 Phosphorylation by the Disulfiram/Copper Complex in Oral Cancer Cells

**DOI:** 10.3390/ijms23169137

**Published:** 2022-08-15

**Authors:** Gunng-Shinng Chen, Ssu-Yu Chen, Shu-Ting Liu, Cheng-Chih Hsieh, Shiao-Pieng Lee, Shih-Ming Huang

**Affiliations:** 1 School of Dentistry, Department of Dentistry of Tri-Service General Hospital, National Defense Medical Center, Taipei City 114, Taiwan; 2 Department of Biochemistry, National Defense Medical Center, Taipei City 114, Taiwan; 3 School of Pharmacy and Institute of Pharmacy, National Defense Medical Center, Taipei City 114, Taiwan; 4 Department of Pharmacy, Kaohsiung Veterans General Hospital, Kaohsiung 813, Taiwan

**Keywords:** c-Myc, disulfiram, reactive oxygen species, hypoxia inducible factor 1α

## Abstract

MYC has a short half-life that is tightly regulated through phosphorylation and proteasomal degradation. Many studies have claimed that treatment with disulfiram (DSF) with or without copper ions can cause cancer cell death in a reactive oxygen species (ROS)-dependent manner in cancer cells. Our previous study showed that the levels of c-Myc protein and the phosphorylation of threonine 58 (T58) and serine 62 (S62) increased in DSF-Cu-complex-treated oral epidermoid carcinoma Meng-1 (OECM-1) cells. These abovementioned patterns were suppressed by pretreatment with an ROS scavenger, N-acetyl cysteine. The overexpression of c-Myc failed to induce hypoxia-inducible factor 1α protein expression, which was stabilized by the DSF-Cu complex. In this study, we further examined the regulatory mechanism behind the induction of the c-Myc of the DSF-Cu complex in an OECM-1 cell compared with a Smulow–Glickman (SG) human normal gingival epithelial cell. Our data showed that the downregulation of c-Myc truncated nick and p62 and the induction of the ratio of H3P/H3 and p-ERK/ERK might not be involved in the increase in the amount of c-Myc via the DSF/copper complexes in OECM-1 cells. Combined with the inhibitors for various signaling pathways and cycloheximde treatment, the increase in the amount of c-Myc with the DSF/copper complexes might be mediated through the increase in the stabilities of c-Myc (T58) and c-Myc (S62) proteins in OECM-1 cells. In SG cells, only the c-Myc (T58) protein was stabilized by the DSF-Cu (I and II) complexes. Hence, our findings could provide novel regulatory insights into the phosphorylation-dependent stability of c-Myc in DSF/copper-complex-treated oral squamous cell carcinoma.

## 1. Introduction

c-Myc is a transcription factor that regulates apoptosis, cellular growth, differentiation, and metabolism via a protein–protein network to activate or repress the transcription of its target genes [1,2,3,4]. The MYC protein is composed of five major domains: MYC boxes I and II (MBI and MBII), the transactivation domain at the N-terminus, the nuclear localization signal, and basic helix–loop–helix and leucine zipper (bHLH and LZ) domains at the C-terminus. This C-terminal domain also allows MYC to heterodimerize with its binding partner MAX and associate with E-box DNA sequences (CACGTG), which is essential for its transcriptional and transforming activity [3]. MYC is a short-half-life protein that is tightly regulated through phosphorylation and proteasomal degradation [5].

Normal cycling cells regulate the levels of MYC by tightly controlling its transcription, translation, and protein stability [4,6,7]. One mechanism regulating MYC protein stability includes the sequential phosphorylation of two highly conserved phosphorylation sites at residues serine 62 (S62) and threonine 58 (T58) [5,6]. In response to growth signals, MYC is first phosphorylated at S62 by one of several proline-directed kinases including extracellular-signal-regulated kinases (ERKs) or cyclin-dependent kinases, which transiently increases MYC stability. Subsequently, the phosphorylation at T58 is mediated by the processive kinase glycogen synthase kinase-3β (GSK3β) or bromodomain-containing protein 4, initiating the dephosphorylation of S62 by protein phosphatase 2A (PP2A) and the ubiquitination of MYC by the E3 ligase complex SCF-FBW7 for proteasome degradation.

Tumors with stable MYC expression have elevated levels of phospho-S62 (p-S62)-MYC and low levels of phospho-T58 (p-T58)-MYC [6,8]. Mitogenic pathways, such as RAS–MEK–ERK signaling, can increase p-S62 levels and, thereby, increase MYC stability. A cytoplasmic form of c-Myc, Myc-nick, is generated by calpain-dependent proteolysis at lysine 298 [9,10]. Myc-nick retains the conserved Myc box regions but lacks the nuclear localization signals and the bHLH-LZ domain essential for heterodimerization with Max and DNA binding. Myc-nick induces alpha-tubulin acetylation and altered cell morphology by recruiting histone acetyltransferase GCN5 to microtubules. A recent study showed that MYC-nick prevented TNF-induced necroptosis [11].

MYC is a key driver of cell-cycle entry, and one of its many functions in this context is to promote mitochondrial biogenesis in preparation for cell division and oxidative metabolism as a source of energy and chemical intermediates for biosynthesis [4,7,12]. Myc also directs the substrate shift by transactivating multiple glycolytic enzymes and downregulating fatty acid oxidation genes by inhibiting peroxisome proliferator-receptor gamma coactivator 1-alpha (PGC-1α) expression. The negative regulation of PGC-1α by MYC occurs at a post-transcriptional level, but the mechanism is unknown. The effects of MYC on mitochondrial biogenesis can be neutralized by the co-expression of hypoxia-inducible factors (HIFs) [13,14]. HIFs are positive regulators of Mxi1, a MYC-related transcription factor, which competes for binding to Max [13]. Under extreme hypoxic conditions, HIFs promote the ubiquitin-dependent proteolysis of MYC [15].

The interplay between genetic mutations and altered levels of transcripts, proteins, and metabolites results in the complex of oral squamous cell carcinoma (OSCC) molecular pathogenesis [16,17]. Alcohol intake, especially aldehyde, is an important risk factor for the development of OSCC. Disulfiram (DSF) is the best-known aldehyde dehydrogenase (ALDH) irreversible inhibitor [18,19], proteasome inhibitor, cancer-associated pathway suppressor [20,21,22], and metal chelator [23]. Superfluous copper ions may displace other metals from their cognate ligands in many important enzymes, such as specific prolyl hydroxylases (PHDs) for HIF-1α protein degradation, resulting in the impairment of enzymatic activities [24]. Similar to the competition of Fe^2+^ (a cofactor for PHD activity) by copper ions, reactive oxygen species (ROS) alter the oxidation state of Fe^2+^ to Fe^3+^, which cannot be utilized, consequently promoting HIF-1α stabilization [25].

Repurposing an old drug for a new application in cancer therapy is an attractive field [26]. DSF, an anti-alcoholism drug, is a potential drug against cancer, which suppresses different cancer-associated pathways including ROS, ALDH, and others [18,19,20,21,22,27]. Current therapeutic DSF serves as a metal chelator, which is primarily complexed with Cu^2+^. In our previous study, we complexed DSF with CuCl or CuCl_2_ to examine their differences on OECM-1 cells [28]. Our findings showed that the primary anti-tumor activity from the DSF-CuCl_2_ complex included cell cycle, cell proliferation, senescence, ROS, mitochondrial dysfunctions, ROS-induced HIF-1α, and c-Myc expression. The DSF-CuCl_2_ complex failed to inhibit ALDH enzyme activity in OECM-1 but not SG cells. The functional interaction between c-Myc and HIF-1α for the stability of c-Myc in OSCC is a puzzle.

In this study, we further analyzed the stability mechanisms of c-Myc involved in the phosphorylation status of residues threonine 58 and serine 62 and its potential crosstalk with HIF-1α in OSCC treated with the DSF/copper complex. We hope that our findings will provide novel regulatory insights into the functional role of c-Myc with the DSF/copper complex in OSCC.

## 2. Results

### 2.1. The Signaling Pathways Were Involved in the Regulation of c-Myc by the DSF/Copper Complex in the OECM-1 and SG Cells

**Figure 1:**
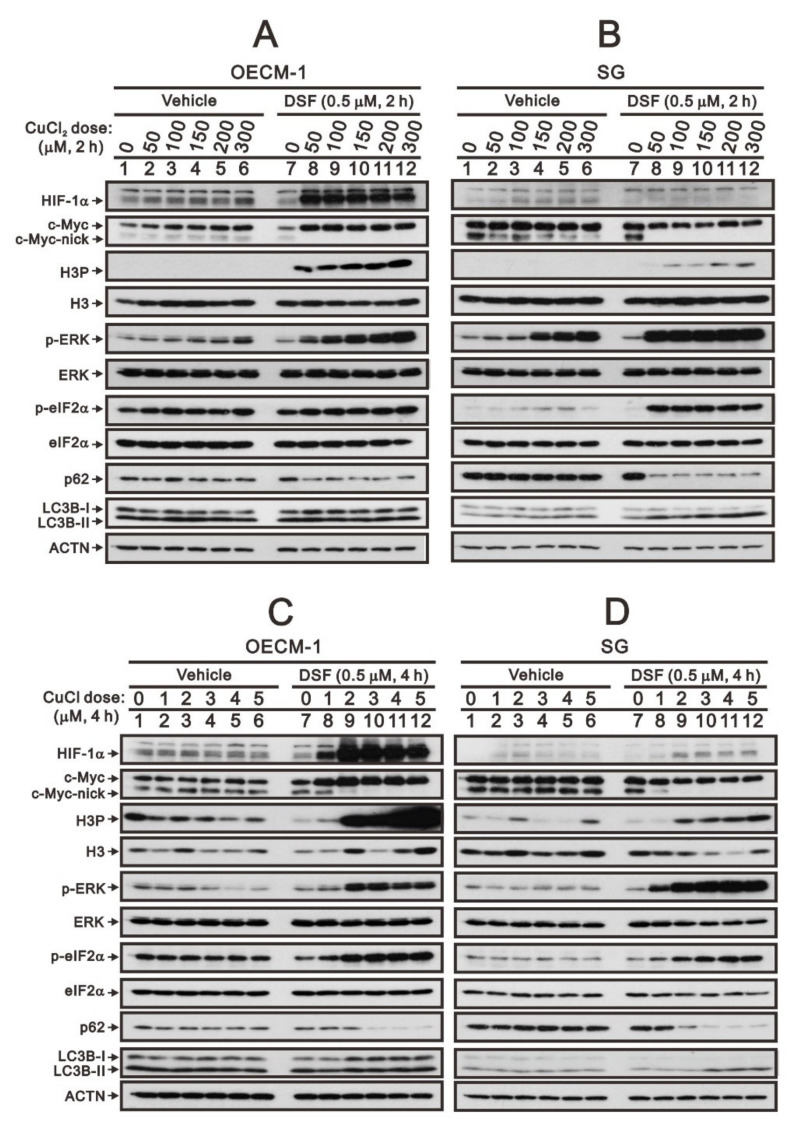
The effects of DSF, copper ions, and DSF/copper complexes on c-Myc and HIF-1α proteins in OECM-1 and SG cells. OECM-1 (**A**,**C**) and SG (**B**,**D**) cells were treated with the indicated concentrations of copper ions in the absence or presence of DSF for 2 h (**A**,**B**) or 4 h (**C**,**D**). The cell lysates were subjected to Western blot analysis using antibodies against the indicated proteins. ACTN was a loading protein control. The results are representative of three independent experiments.

Based on our previous study [28], we reconfirmed that the DSF/copper complex dramatically induced HIF-1α expression, accompanied by an increase in full-length c-Myc expression and a decrease in truncated c-Myc and Myc-nick in OECM-1 cells (Figure 1A,C). In SG cells, the DSF/copper complex failed to induce the expression of HIF-1α, decreased the level of Myc-nick, and mildly decreased the level of full-length c-Myc expression (Figure 1B,D). Tumors with stable MYC expression have elevated levels of p-S62 and low levels of p-T58 via mitogenic pathways, such as RAS–MEK–ERK signaling [6,8]. In addition, c-Myc might be regulated in the propagation of cell cycle, endoplasmic reticulum (ER) stress, and autophagy [4,7,12]. Hence, we examined the relationships of H3P (phosphorylated histone H3 serine 10)-histone H3 (cell cycle G2/M), p-ERK/ERK (mitogenic pathway), p-eIF2α/eIF2α (ER stress), LC3B/p62 (autophagy) with the levels of c-Myc in OECM-1 and SG cells. Our Western blotting analysis demonstrated that the DSF/copper complex induced higher phosphorylation levels of H3P in OECM-1 cells and induced higher phosphorylation levels of ERK and eIF2α in SG cells. The DSF complexed with Cu^2+^ or Cu^+^ had similar effects on the abovementioned protein expressions (Figure 1). The effect of Cu^2+^ alone had inductive effects on HIF-1α, c-Myc, p-ERK, and p-eIF2α in OECM-1 cells and HIF-1α, p-ERK, and p-eIF2α in SG cells. The effect of Cu^+^ alone had suppressive effects on H3P, p-ERK, and p-eIF2α in OECM-1 cells and p-eIF2α in SG cells and inductive effects on H3P in SG cells.

**Figure 2:**
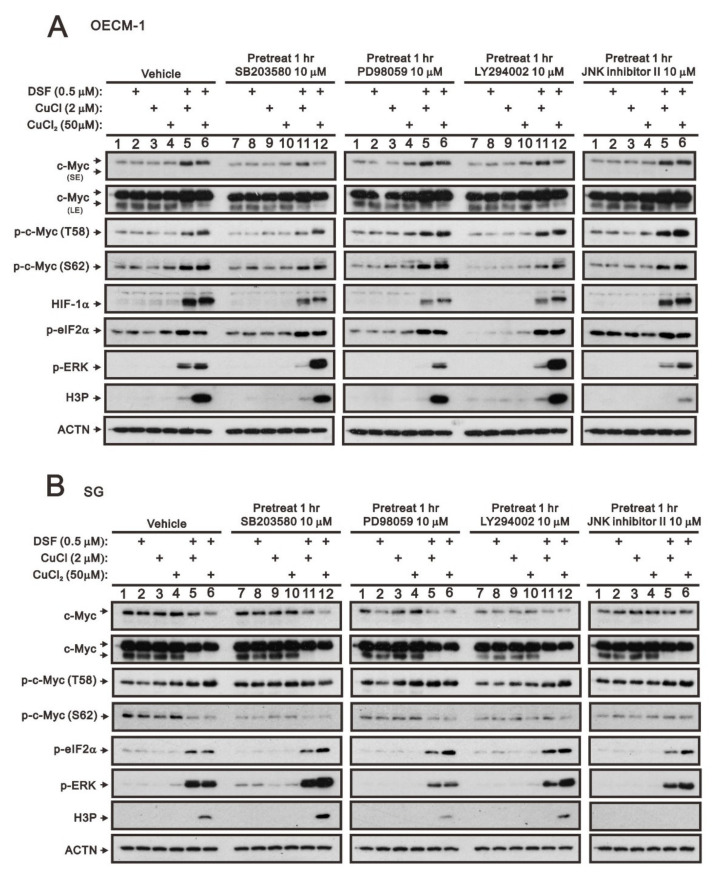
The effects of DSF, copper ions, and DSF/copper complexes on the signaling pathways involved in the c-Myc and HIF-1α proteins in OECM-1 and SG cells. (**A**) OECM-1 and (**B**) SG cells were pretreated with the indicated 10 μM of signaling inhibitors for 1 h and then treated with 2 μM of CuCl or 50 μM of CuCl_2_ plus 0.5 μM of DSF for 2 h (added indicated drugs labeled with +). The cell lysates were subjected to Western blot analysis using antibodies against the indicated proteins. ACTN was a loading protein control. The results are representative of three independent experiments.

The entire regulatory cycle of c-Myc from signal transduction events leads to gene expression, to stabilization, and to degradation [4,6,7]. The dual phosphorylation of c-Myc, T58, and S62 is an important signal for the stability of c-Myc [5,6]. Mitogenic pathways, including p38, MEK/ERK, PI3K, and JNK signaling pathways, might increase the p-S62 levels and, thereby, increase MYC stability. We used four kinase inhibitors, SB203580 (p38 MAPK inhibitor), PD98059 (MEK/ERK inhibitor), LY294002 (PI3K inhibitor), and the JNK inhibitor II to examine which kind of kinases were involved in the dual phosphorylation of c-Myc, T58, and S62 in OECM-1 and SG cells. In OECM-1 cells (Figure 2A), SB203580 (p38 MAPK inhibitor) and LY294002 (PI3K inhibitor) suppressed the induction of c-Myc through the DSF/copper complex via the decreasing level of S62 phosphorylation. The JNK inhibitor II elevated the T58 phosphorylation via the DSF/copper complex. We observed that SB203580 (p38 MAPK inhibitor), PD98059 (MEK/ERK inhibitor), and LY294002 (PI3K inhibitor) downregulated the HIF-1α induced by the DSF/copper complex. SB203580 (p38 MAPK inhibitor), PD98059 (MEK/ERK inhibitor), LY294002 (PI3K inhibitor), and JNK inhibitor II had no apparent effect on p-eIF2α. The inductions of p-ERK via the DSF/copper (I) complex were suppressed by SB203580 (p38 MAPK inhibitor), PD98059 (MEK/ERK inhibitor), LY294002 (PI3K inhibitor), and the JNK inhibitor II, but the DSF/copper (II) complex was enhanced by SB203580 (p38 MAPK inhibitor) and LY294002 (PI3K inhibitor). The level of H3P was suppressed by JNK inhibitor II.

In addition to the effect on the level of p-eIF2α, these four kinase inhibitors had different effects on c-Myc, p-ERK, and H3P in SG cells (Figure 2B). The inductions of p-ERK via the DSF/copper (I) complex were suppressed by PD98059 (MEK/ERK inhibitor), LY294002 (PI3K inhibitor), and JNK inhibitor II, but the DSF/copper (II) complex was enhanced by SB203580 (p38 MAPK inhibitor) and LY294002 (PI3K inhibitor) and suppressed by PD98059 (MEK/ERK inhibitor). The level of H3P was suppressed by PD98059 (MEK/ERK inhibitor) and = JNK inhibitor II and enhanced by SB203580 (p38 MAPK inhibitor).

### 2.2. The Protein Stability of c-Myc Was Modulated by the DSF/Copper Complex in the OECM-1 and SG Cells

**Figure 3:**
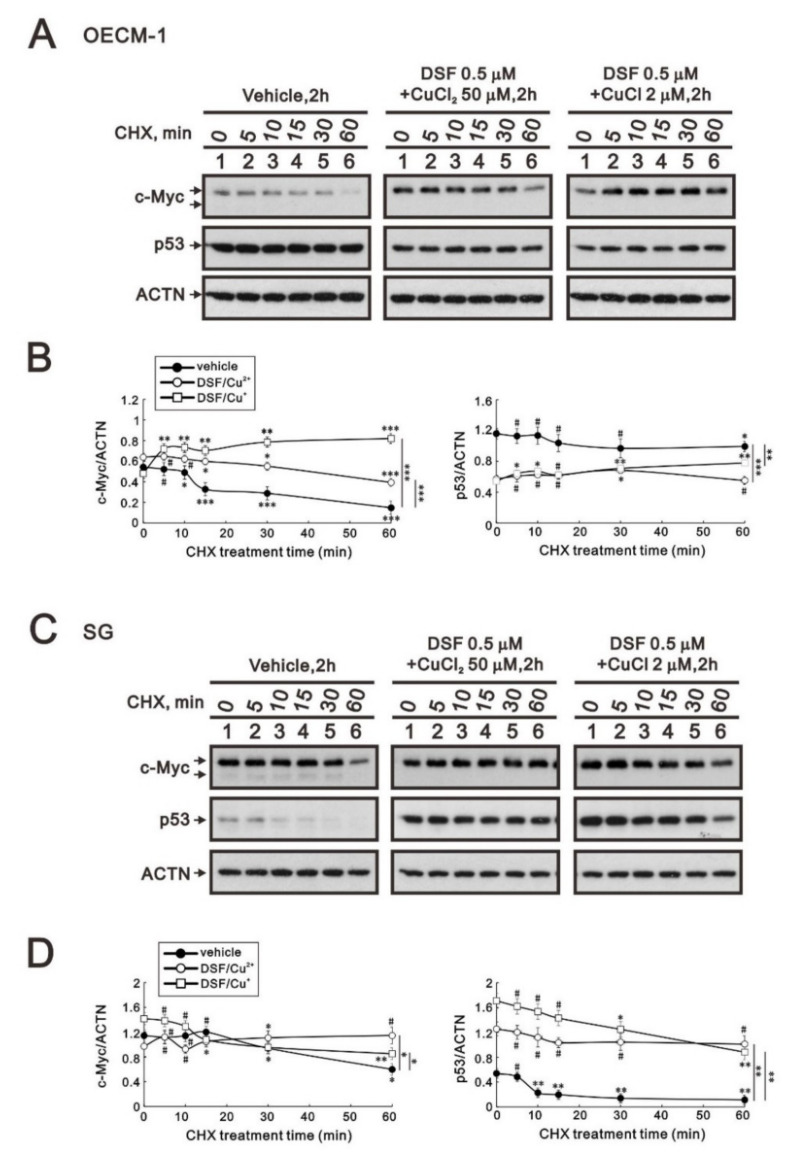
The effects of DSF, copper ions, and DSF/copper complexes on the stability of the c-Myc and p53 proteins in OECM-1 and SG cells. OECM-1 (**A**) and SG (**C**) cells were treated with vehicle, 50 μM of CuCl_2_ plus 0.5 μM of DSF, or 2 μM of CuCl plus 0.5 μM of DSF for 2 h, accompanied by 50 mg/mL CHX for 0, 5, 10, 15, 30, and 60 min. The cell lysates were subjected to Western blot analysis using antibodies against c-Myc and p53 proteins. ACTN was a loading protein control. The protein bands were quantified through pixel density scanning and evaluated using ImageJ, version 1.44a (http://imagej.nih.gov/ij/) (accessed on 1 May 2022). The ratios of c-Myc/ACTN and p53/ACTN were plotted in OECM-1 (**B**) and SG (**D**) cells. The results are representative of three independent experiments. The quantified protein bands were compared in cells treated with indicated time of CHX to the 0 h. # *p* > 0.05, * *p* < 0.05, ** *p* < 0.01, and *** *p* < 0.001.

Many studies have demonstrated that c-MYC, p53, and HIF-1α are crucial for tumor cells’ aberrant metabolic behavior [29]. In addition, p53 is one of the target genes of c-Myc. Here, we applied a de novo protein synthesis inhibitor, cycloheximide (CHX), to examine the effects of the two DSF/copper complexes on c-Myc and the p53 protein. The c-Myc proteins were increased by these two DSF/copper complexes (*p* = 0.002 for CuCl_2_ and 0.04 for CuCl compared at the time of no CHX treatment), but only the DSF/Cu^+^ complex extended its stability in OECM-1 cells (Figure 3A). In the SG cells, the two DSF/copper complexes had no effect on the c-Myc proteins, but only the DSF/Cu^2+^ complex extended its stability (Figure 3B). The amounts of p53 protein were downregulated by these two DSF/copper complexes (*p* = 0.001 for CuCl_2_ and 0.001 for CuCl compared at the time of no CHX treatment), which had no effect on its stability in OECM-1 cells (Figure 3A), whereas in SG cells, the amounts of p53 proteins were enhanced by these two DSF/copper complexes (*p* = 0.003 for CuCl_2_ and 0.005 for CuCl compared at the time of no CHX treatment), and the DSF/Cu^2+^ complex further extended its stability (Figure 3B).

### 2.3. The Involvement of Cytosolic and Mitochondrial ROS Generation in the Regulation of c-Myc by the DSF/Copper Complex in the OECM-1 and SG Cells

**Figure 4:**
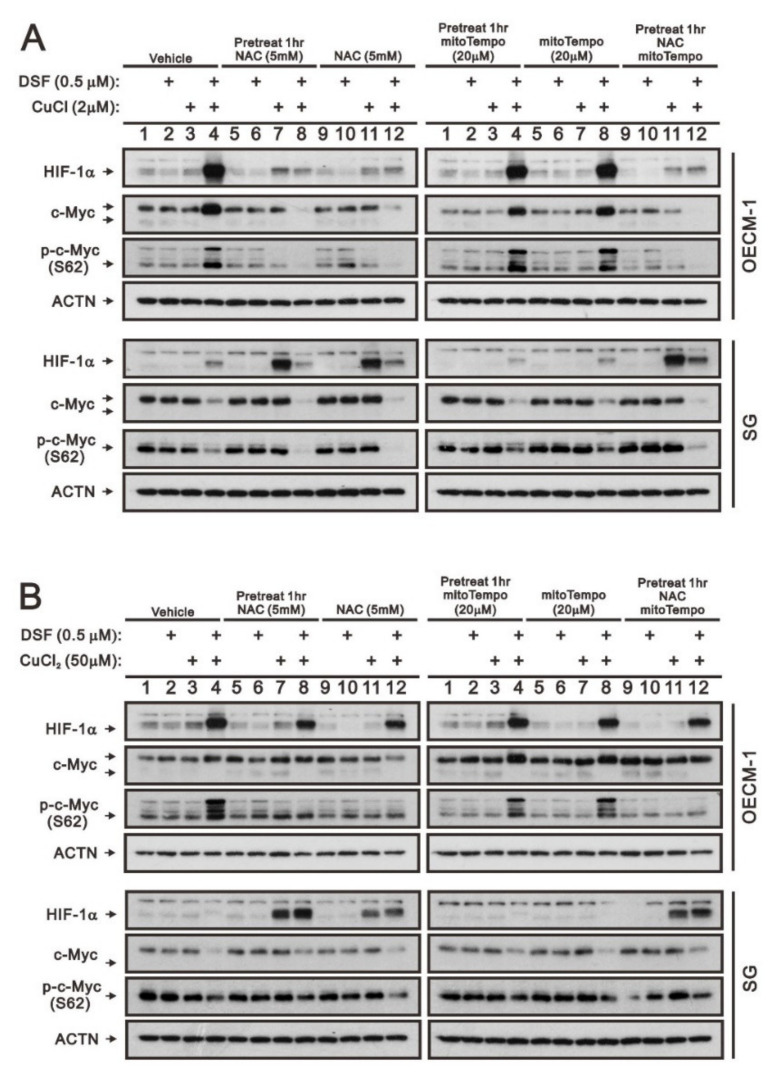
The effects of DSF, copper ions, and DSF/copper complexes on the c-Myc and HIF-1α proteins by NAC and mitoTEMPO in OECM-1 and SG cells. OECM-1 and SG cells were treated with (**A**) 2 μM of CuCl plus 0.5 μM of DSF and (**B**) 50 μM of CuCl_2_ plus 0.5 μM of DSF for 2 h (added indicated drugs labeled with +) in the presence of pretreated NAC, mitoTEMPO, or co-treated NAC with mitoTEMPO for 1 h. The cell lysates were subjected to Western blot analysis using antibodies against the indicated proteins. ACTN was a loading protein control. The results are representative of three independent experiments.

Our previous work revealed that ROS play an important role in the induction of c-Myc and HIF-1α in OECM-1 cells [28]. Here, we further examined the different functions between cytosolic and mitochondrial ROS using two respective ROS scavengers, NAC and mitoTempo, in OECM-1 and SG cells. HIF-1α, full-length c-Myc, and p-c-Myc (S62) proteins induced by the DSF/Cu^+^ complex were suppressed by NAC whether pretreated or not, but they were not disrupted by mitoTempo in OECM-1 cells (Figure 4A). OECM-1 cells treated with the DSF/Cu^2+^ complex demonstrated that NAC and mitoTempo had no effect on the induction of HIF-1α, but NAC suppressed the induction of p-c-Myc (S62), non-pretreated NAC suppressed the induction of full-length c-Myc, and mitoTempo enhanced the induction of full-length c-Myc (Figure 4B). Interestingly, NAC induced the HIF-1α proteins in SG cells treated with CuCl, CuCl_2_, and DSF/Cu^2+^ (Figure 4A,B).

### 2.4. The Involvement of GSK3β Activity in the Regulation of c-Myc by the DSF/Copper Complex in the OECM-1 and SG Cells

**Figure 5:**
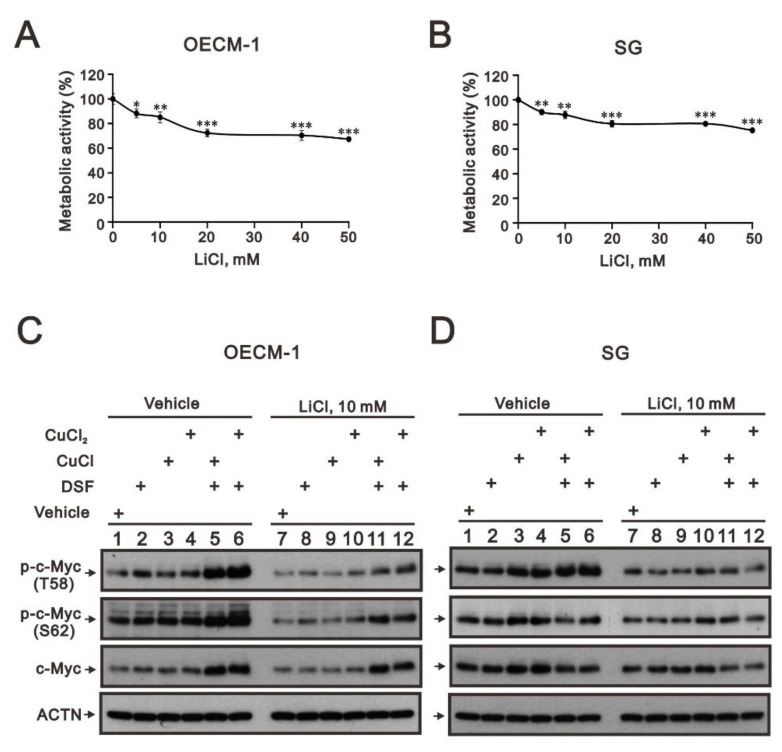
The effects of LiCl on the c-Myc proteins in DSF/copper-complex-treated OECM-1 and SG cells. (**A**) OECM-1 and (**B**) SG cells were treated with 0, 5, 10, 20, 40, and 50 mM LiCl for 24 h. The metabolic activity was measured using MTT assays. * *p* < 0.05, ** *p* < 0.01, and *** *p* < 0.001. (**C**) OECM-1 and (**D**) SG cells were pretreated with 10 mM LiCl for 1 h and then treated with 50 μM of CuCl_2_ plus 0.5 μM of DSF or 2 μM of CuCl plus 0.5 μM of DSF for 2 h (added indicated drugs labeled with +). The cell lysates were subjected to Western blot analysis using antibodies against the indicated proteins. ACTN was a loading protein control.

**Figure 6:**
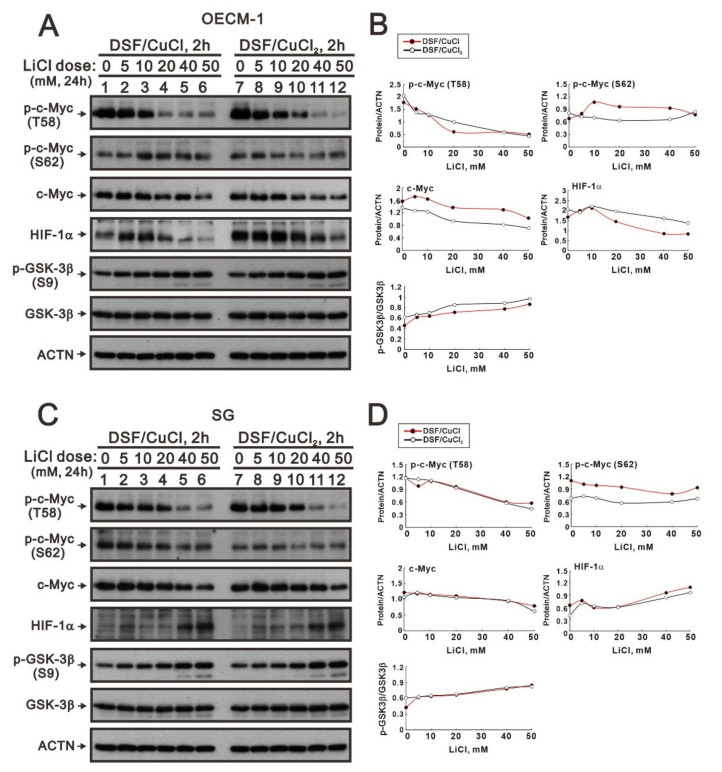
The effects of DSF, copper ions, and DSF/copper complexes on the c-Myc and HIF-1α proteins in LiCl-treated OECM-1 and SG cells. (**A**) OECM-1 and (**C**) SG cells were treated with 50 μM of CuCl_2_ plus 0.5 μM of DSF or 2 μM of CuCl plus 0.5 μM of DSF for 2 h, accompanied by 0, 5, 10, 20, 40, and 50 mM LiCl for 24 h. The cell lysates were subjected to Western blot analysis using antibodies against the indicated proteins. ACTN was a loading protein control. The protein bands (**A**,**C**) were quantified through pixel density scanning and evaluated using ImageJ, version 1.44a (http://imagej.nih.gov/ij/) (accessed on 1 May 2022). The ratios of protein/ACTN and pGSK3β/GSK3β were plotted, and red lines are for the DSF/CuCl complexes and black lines for the DSF/CuCl_2_ complexes in (**B**) OECM-1 and (**D**) SG cells.

The phosphorylation at T58 of c-Myc is mediated by the processive kinase GSK3β [5,6]. Lithium, a medication for bipolar disorder, is a direct and indirect inhibitor of GSK-3 [30]. Lithium chloride (LiCl) had similar cytotoxic effects, and a relatively safe dosage below 20 mM retained 80% of activities in OECM-1 and SG cells (Figure 5A,B). We next examined the effect of LiCl alone or combined with DSF/copper complexes for wild-type and phosphoarylated c-Myc proteins in OECM-1 and SG cells (Figure 5C,D). In general, LiCl suppressed the basal levels and the DSF/copper complexes induced wild-type and phosphoarylated c-Myc proteins in OECM-1 and SG cells. Hence, we treated OECM-1 and SG cells with 0, 5, 10, 20, 40, and 50 mM LiCl for 24 h and analyzed the cells using the Western blot analysis (Figure 6A,C). Our Western blot data demonstrated that lithium chloride elevated the inhibitory p-GSK-3β (S9) and decreased the levels of full-length c-Myc and p-c-Myc (T58) proteins in a dose-dependent manner in OECM-1 and SG cells (Figure 6B,D). Lithium chloride elevated the level of the p-c-Myc (S62) protein in OECM-1 cells but decreased the level in SG cells. The induction of HIF-1α by the DSF-Cu (I and II) complex was suppressed by lithium chloride in a dose-dependent manner (Figure 6B), whereas a higher dosage of lithium chloride induced HIF-1α by the DSF-Cu (I and II) complex in SG cells (Figure 6D).

**Figure 7:**
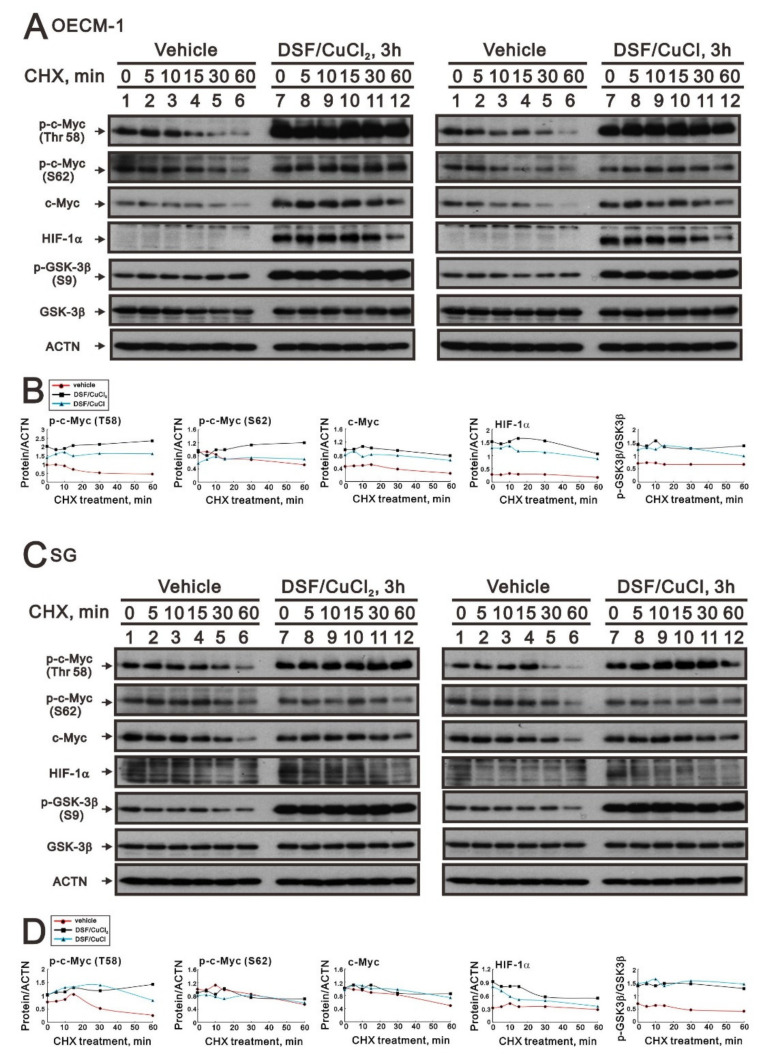
The effects of DSF, copper ions, and DSF/copper complexes on GSK3β activity for the c-Myc and HIF-1α proteins in OECM-1 and SG cells. (**A**) OECM-1 and (**C**) SG cells were treated with 50 μM of CuCl_2_ plus 0.5 μM of DS or 2 μM of CuCl plus 0.5 μM of DSF for 3 h, accompanied by 50 mg/mL CHX for 0, 5, 10, 15, 30, and 60 min. The cell lysates were subjected to Western blot analysis using antibodies against the indicated proteins. ACTN was a loading protein control. The protein bands (**A**,**C**) were quantified through pixel density scanning and evaluated using ImageJ, version 1.44a (http://imagej.nih.gov/ij/) (accessed on 1 May 2022). The ratios of protein/ACTN and pGSK3β/GSK3β are plotted with red lines (vehicle), mint green lines (DSF/CuCl), and black lines (DSF/CuCl_2_) in (**B**) OECM-1 and (**D**) SG cells.

We further examined the effect of the DSF-Cu (I and II) complex on the stability of full-length c-Myc, p-c-Myc (T58), and p-c-Myc (S62) proteins using the CHX treatment in OECM-1 and SG cells (Figure 7A,C). We first observed the induction of p-GSK-3β (S9) via the DSF/copper complex in OECM-1 and SG cells (Figure 6B,D). Next, the DSF/copper complex, Cu(II) or Cu(I), induced full-length c-Myc, p-c-Myc (T58), and p-c-Myc (S62) proteins’ expression and enhanced their stabilities in OECM-1 cells (Figure 7B). In SG cells, only the p-c-Myc (T58) protein was induced and stabilized by the DSF/copper complex, Cu(II) and Cu(I); however, it had a repressive effect on the full-length c-Myc and p-c-Myc (S62) proteins (Figure 7D). The induction of HIF-1α via the DSF/copper complex was parallel with the induction of the full-length c-Myc in OECM-1 cells. In SG cells, the induction of HIF-1α via the DSF-Cu (I and II) complex was relatively unstable.

## 3. Discussion

Genetic alterations affecting *MYC* proto-oncogenes and MYC-related signaling pathways are among the most common in human cancers [1,2,3,4]. The entire regulatory cycle of c-Myc from signal transduction events leads to gene expression, to stabilization, and to degradation [4,6,7]. Oncogenic MYC also retains the conflicting functions of driving proliferation and cell death [31]. Common anti-cancer drugs (e.g., chemotherapeutics) often trigger both apoptotic pathways, thereby increasing their reliance on MYC. The dual phosphorylated form of c-Myc (T58 and S62) has been recognized, with further dephosphorylated S62, degraded by the 26S proteasome. Our previous study showed that the primary anti-tumor activity from the DSF-Cu complex included cell cycle, cell proliferation, senescence, ROS, mitochondrial dysfunctions, ROS-induced HIF-1α, and c-Myc expression [28]. In this study, we further determined the regulatory mechanism(s) of c-Myc expression via the DSF-Cu complex in OECM-1 cells compared with SG cells. Our data showed that the downregulation of c-Myc truncated nick and p62 and the induction of the ratio of H3P/H3. p-ERK/ERK might not be involved in the increased amount of c-Myc through DSF/copper complexes in OECM-1 cells. Combined with the inhibitors for various signaling pathways and CHX treatment, the increasing amount of c-Myc via DSF/copper complexes might be mediated through the increasing stabilities of c-Myc (T58) and c-Myc (S62) proteins in OECM-1 cells. In SG cells, only the c-Myc (T58) protein was stabilized by the DSF-Cu (I and II) complexes.

DSF, an anti-alcoholism drug, is a potential drug against cancer as it can suppress different cancer-associated pathways including ROS, ALDH, and others [18,19,20,21,22]. Our previous data suggested that ALDH activity was functional in SG cells, and the anti-tumor activity of the DSF-Cu complex in OECM-1 cells might be beyond the inhibitor for ALDH activity [28]. The pretreatment of NAC or NAC treatment suppressed the DSF/Cu^+^ complex induction of c-Myc, c-Myc (S62), and HIF-1α and the DSF/Cu^2+^ complex induction of c-Myc and c-Myc (S62) in OECM-1 cells, and it further induced HIF-1α by Cu^+^, Cu^2+^, and DSF/Cu^2+^ in SG cells. In contrast to the cytosolic ROS scavenger NAC, the mitochondrial ROS scavenger mitoTempo had no suppressive or inductive effect on c-Myc, c-Myc (S62), or the HIF-1α protein in OECM-1 and SG cells. Our previous study demonstrated that the DSF-CuCl_2_ complex had better anti-tumor activity than the DSF-CuCl complex in OECM-1 cells, and the DSF-CuCl complex had apparent effects on SG cells. Copper ions may displace ferrous iron from their cognate ligands in PHDs for HIF-1α protein degradation. Here, the DSF-Cu (I and II) complex significantly stabilized the HIF-1α protein in OECM-1 cells and not SG cells. In SG cells, Cu^+^, Cu^2+^, and DSF/Cu^2+^ stabilized the HIF-1α proteins in the presence of the cytosolic ROS scavenger NAC, and the DSF-Cu (I and II) complex significantly stabilized the HIF-1α protein in the presence of the LiCl concentration over 40 mM.

In mammalian cells, upon stimulation with insulin or other growth factors, GSK-3 is rapidly phosphorylated at serine 21 in GSK-3α or serine 9 in GSK-3β, resulting in the inhibition of GSK-3 kinase activity [32]. GSK-3 activity suppresses cell proliferation and survival. Lithium, a medication for bipolar disorder, is a direct and indirect inhibitor of GSK-3 [30]. Many studies have shown that the phosphorylation at T58 of c-Myc is mediated by the processive kinase GSK3β [33,34,35]. The DSF-Cu (I and II) complex dramatically induced the phosphorylation of GSK-3β (S9) and c-Myc (T58) in the OECM-1 and SG cells (Figure 6), whereas for the induction of p-GSK-3β (S9) by LiCl, the amounts of c-Myc and p-c-Myc (T58) were suppressed in a LiCl dose-dependent manner in OECM-1 and SG cells (Figure 5 and Figure 6). The effect of LiCl on the amount of HIF-1α protein was different between the OECM-1 and SG cells. The effect of LiCl-treated OECM-1 cells on the HIF-1α protein might be consistent with the study of Dr. Kietzmann’s laboratory which suggested that GSK3β phosphorylates HIF-1α and mediates its destabilization [36]. It would be interesting to determine why LiCl stabilizes the HIF-1α protein in SG cells. Combined with the current findings of the phosphorylation status of the c-Myc residues at T58 and S62, this suggests that our previous data showing that there was no effect on the HIF-1α gene or protein from overexpressing wild-type c-Myc might not lead to a complete conclusion.

Our previous study demonstrated that the DSF-Cu (I and II) complex significantly induced c-Myc transcription, translation, the phosphorylation of T58 and S62, and protein stability or suppressed the nick form of c-Myc via mediation through post-translation modification [28]. The full-length c-Myc was degraded by the 26S proteasome complex when the dual phosphorylated form of c-Myc (T58 and S62) was recognized, and PP2A further dephosphorylated S62. In this study, we proposed that the increasing half-life of the c-Myc (S62) protein was the key aspect in determining the stabilization of the full-length c-Myc via the DSF-Cu (I and II) complex in OECM-1. Our signaling pathway analysis showed that p38 and PI3K inhibitors suppressed the DSF-Cu (I and II) complex in the induction of c-Myc (S62) and HIF-1α proteins, consistently supported by the fact that PP2A activity is modulated by some kinases, ROS, and the HIF-1α protein [37]. The cancerous inhibitor of PP2A interacts with some components of PP2A to prevent holoenzyme formation and the dephosphorylation of c-Myc (S62) [38]. Hence, the PP2A system might be regulated by the current DSF-Cu (I and II) complex mediating through the endogenous PP2A inhibitor, ROS, or protein–protein interaction via HIF-1α. In addition to a proteasome inhibitor, the DSF-Cu (I and II) complex might decrease the dominant-negative effect of MYC-nick to elevate its cytotoxicity in oral cancers. These provide new avenues by which to address the degradation mechanism for the DSF-Cu (I and II) complex in oral cancers.

## 4. Materials and Methods

### 4.1. Cell Culture and Chemicals

OECM-1 and SG cells were cultured in Roswell Park Memorial Institute (RPMI) 1640 (Corning, Corning City, NY, USA) containing 10% fetal bovine serum (FBS) and 1% penicillin–streptomycin (Thermo Fisher Scientific, Waltham, MA, USA). *N*-acetyl cysteine (NAC), copper (II) chloride, MitoTempo, and thiazolyl blue tetrazolium bromide (MTT) were obtained from Sigma Aldrich (Burlington, MA, USA). Copper (I) chloride was obtained from Alfa Aesar (Wardhill, MA, USA).

### 4.2. Metabolic Activity Analysis

The 6 × 10^4^ OECM-1 and SG cells were plated in 24-well culture plates and cultured for the indicated drug treatment. The cells were then incubated with 0.5 mg/mL MTT solution for 1 h at 37 °C. Dimethyl sulfoxide (DMSO; 200 μL) was then added, and the absorbances at 570 nm and 650 nm were measured using an ELISA plate reader (Multiskan EX, Thermo Fisher Scientific, Waltham, MA, USA). The control group containing cells cultured in medium only was defined as 100% metabolic activity.

### 4.3. Western Blotting

For Western blot analysis, the day before treatment, 3 × 10^5^ SG and OECM-1 cells were seeded into 6-well culture plates, and the cells were grown to 70–80% confluency and treated with the indicated treatments. We used filtered ddH_2_O as the vehicle control. After treatment, cells were lysed with lysis buffer (100 mM Tris-HCl (pH 8.0), 150 mM NaCl, 0.1% SDS, and 1% Triton X-100). Protein lysates were prepared with 4× protein loading dye and denatured at 95 °C for 10 min, separated on 10% SDS-PAGE and blotted onto PVDF membranes. The membranes were blocked in TBST buffer (50 mM Tris-HCl (pH 8.0), 150 mM NaCl, 0.1% Tween 20, and 5% nonfat dry milk) for 1 h. The primary antibodies used, α-actinin (ACTN) (H-2) catalog no. 17829 (1:5000 dilution), anti-p53 (DO-1) catalog no. 126 (1:1000 dilution), anti-p62 (D-3) catalog no. 28359 (1:1000 dilution), anti-eIF-2a catalog no. 9722 (1:1000 dilution), anti-p-eIF-2α catalog no. 9721 (1:1000 dilution), anti-ERK catalog no. 4695 (1:1000 dilution), anti-p-ERK catalog no. 4370 (1:1000 dilution), anti-GSK3β catalog no. 12456 (1:1000 dilution), anti-p-GSK3b (S9) catalog no. 5558 (1:1000 dilution), anti-HIF-1a catalog no. 14179 (1:1000 dilution), anti-histone H3 catalog no. 9715 (1:1000 dilution), anti-H3P (phosphorylated histone H3 serine 10) catalog no. 9701 (1:1000 dilution), anti-LC3B catalog no. 2775 (1:1000 dilution), anti-c-Myc catalog no. 13987 (1:1000 dilution), anti-phospho-c-Myc (S62) catalog no. 13748 (1:1000 dilution), anti-phospho-c-Myc (T58) catalog no. 46650 (1:1000 dilution), and HRP-conjugated secondary antibodies (anti-mouse IgG catalog no. AP192P (1:5000 dilution) and anti-rabbit IgG catalog no. AP132P) (1:5000 dilution), were purchased from Merck-Millipore.

### 4.4. Statistical Analysis

The values are expressed as the mean ± SD of at least three independent experiments. All the comparisons between groups were made using Student’s *t*-tests. The statistical significance was set at *p* < 0.05.

## 5. Conclusions

Combined with the inhibitors for various signaling pathways and cycloheximde treatment, the increasing amount of c-Myc with the DSF/copper complexes might be mediated through the increasing stabilities of c-Myc (T58) and c-Myc (S62) proteins in OECM-1 cells. In SG cells, only the c-Myc (T58) protein was stabilized by the DSF-Cu complexes. Hence, our findings could provide novel regulatory insights into the phosphorylation-dependent stability of c-Myc in DSF/copper-complex-treated oral squamous cell carcinoma.

## Data Availability

The data presented in this study are available on request from the corresponding author.
